# Unlocking the Protective Potential of Upper Respiratory Infection Treatment Histories against Alzheimer’s Disease: A Korean Adult Population Study

**DOI:** 10.3390/jcm13010260

**Published:** 2024-01-02

**Authors:** Ho Suk Kang, Ji Hee Kim, Joo-Hee Kim, Woo Jin Bang, Hyo Geun Choi, Nan Young Kim, Ha Young Park, Mi Jung Kwon

**Affiliations:** 1 Division of Gastroenterology, Department of Internal Medicine, Hallym University Sacred Heart Hospital, Hallym University College of Medicine, Anyang 14068, Republic of Korea; hskang76@hallym.or.kr; 2 Department of Neurosurgery, Hallym University Sacred Heart Hospital, Hallym University College of Medicine, Anyang 14068, Republic of Korea; kimjihee.ns@gmail.com; 3 Division of Pulmonary, Allergy, and Critical Care Medicine, Department of Medicine, Hallym University Sacred Heart Hospital, Hallym University College of Medicine, Anyang 14068, Republic of Korea; luxjhee@gmail.com; 4 Department of Urology, Hallym University Sacred Heart Hospital, Hallym University College of Medicine, Anyang 14068, Republic of Korea; yybbang@hallym.or.kr; 5 Suseo Seoul E.N.T. Clinic and MD Analytics, 10, Bamgogae-ro 1-gil, Gangnam-gu, Seoul 06349, Republic of Korea; mdanalytics@naver.com; 6 Hallym Institute of Translational Genomics and Bioinformatics, Hallym University Medical Center, Anyang 14068, Republic of Korea; honeyny78@gmail.com; 7 Department of Pathology, Busan Paik Hospital, Inje University College of Medicine, Busan 47392, Republic of Korea; hy08.park@gmail.com; 8 Department of Pathology, Hallym University Sacred Heart Hospital, Hallym University College of Medicine, Anyang 14068, Republic of Korea

**Keywords:** Alzheimer’s disease, upper respiratory infection, nested case–control study, national health screening cohort

## Abstract

With increasing interest in the inflammation-pathogen infection hypothesis and its potential links to Alzheimer’s disease (AD) development, there is growing consideration of using upper respiratory infection (URI) treatments as interventions for AD. This nested case–control study explored the potential association between prior URI histories and AD development in a Korean adult population using the national health screening cohort data (2002–2019). The study included 26,920 AD patients and 107,680 matched control individuals, focusing on those seeking respiratory treatment. Logistic regression analyses assessed the impact of URI histories and treatment on AD risk while adjusting for covariates. Our results revealed that over a 1-year period, individuals with URI histories (≥1, ≥2, or ≥3 instances) exhibited decreasing probabilities of developing AD, with risk reductions of 19%, 15%, and 12%, respectively. Expanding our investigation to a 2-year period consistently showed a 17% reduction in AD risk. This effect remained robust across diverse demographic groups and after adjusting for covariates, encompassing comorbidities, hypertension, hyperlipidemia, blood glucose levels, and lifestyle factors. Subgroup analyses further substantiated this association. In conclusion, our findings cautiously suggest a potential protective role of prior URI treatment histories in mitigating the risk of AD development.

## 1. Introduction

Alzheimer’s disease (AD) is a profoundly debilitating neurodegenerative condition that impacts millions of people globally, with its prevalence steadily increasing as populations age [1]. Recent years have witnessed a remarkable rise in the prevalence of AD in many regions, including Korea, where the burden of dementia, including AD, has surged by 30% over the past decade [2]. Despite decades of research, the precise etiology and pathogenesis of AD remain incompletely understood [3]. Traditionally, the amyloid hypothesis has been at the forefront of AD research, focusing on the role of abnormal protein deposits in the brain [4]. It is increasingly acknowledged that AD is a complex condition, shaped by a combination of genetic and environmental factors [3]. In recent years, the emergence of the inflammation–pathogen infection hypothesis has sparked interest in the potential link between infections, particularly upper respiratory infections (URIs), and the onset and advancement of AD [5].

URIs, encompassing a wide spectrum of illnesses such as the common cold, influenza, and sinusitis, are among the most frequent infectious diseases in humans [6]. While the acute symptoms of URIs are typically mild and transient, mounting evidence suggests that they may have more profound and long-lasting effects on human health than previously recognized [6]. Emerging research has implicated inflammatory responses triggered by viral and bacterial pathogens during URIs as potential contributors to neuroinflammation and neuronal damage, processes known to be central in AD pathogenesis [7,8]. Intriguingly, a growing body of research has uncovered connections between bacteria like Chlamydia pneumoniae and viruses like influenza virus, herpes simplex virus, adenoviruses, poliovirus, measles virus, cytomegalovirus, severe acute respiratory syndrome coronavirus 2 (COVID-19), and AD pathology [7,9,10,11], demonstrating that hippocampal cells are highly susceptible to infection by several viruses [12]. In cultured neurons, microbial infections have been observed to induce a notable intracellular buildup of amyloid-β protein, leading to significant disruptions in neuronal function [12]. If the hypothesis suggesting that infections may play a role in the development of AD holds true, addressing these infections could potentially serve as a preventive or therapeutic strategy for AD. These findings have raised the possibility of antiviral treatments as a means to mitigate AD.

Conversely, ongoing clinical trials are currently exploring the potential of antibiotics and antiviral medications in the treatment of individuals with dementia [13,14]. Furthermore, recent experimental research has revealed a close correlation between antimicrobial activity and reduced levels of amyloid-β within the tissue [15].

Leveraging a large, nationally representative health screening cohort, we aimed to address critical questions surrounding the temporal relationship between treatment histories of URIs and AD, the potential role of specific pathogens, and the impact of underlying health conditions. By utilizing robust epidemiological methods and meticulous data analysis, we sought to shed light on whether URI histories could be a modifiable risk factor for AD and provide insights into preventive strategies.

## 2. Patients and Methods

### 2.1. Patient Selection and Data Source

The study utilized data from the Korean National Health Insurance Service—Health Screening Cohort (KNHIS-HSC), a collection of de-identified electronic records specifically designed for research purposes to protect the anonymity of the Korean population, as previously explained [16,17]. Diagnostic codes in this research adhered to the International Classification of Diseases, 10th Revision, Clinical Modification (ICD-10-CM).

This study received approval from the ethics committee at Hallym University (IRB No. 2019-10-023) and was conducted in accordance with the guidelines and regulations of the Institutional Review Board. It did not require written informed consent since it utilized secondary data.

Participants aged 40 and above were initially identified from the dataset, totaling 514,866 individuals with medical claim codes between 2002 and 2019. Of these, 37,427 were AD cases, while 477,439 patients without AD served as controls. To mitigate bias from pre-existing AD, cases diagnosed in 2002–2003 (*n* = 419) and those with missing BMI, fasting blood glucose, or total cholesterol data (*n* = 22) were excluded. Additionally, control participants who had been diagnosed once with specific codes (G30 or F00) were removed (*n* = 7721).

**Figure 1:**
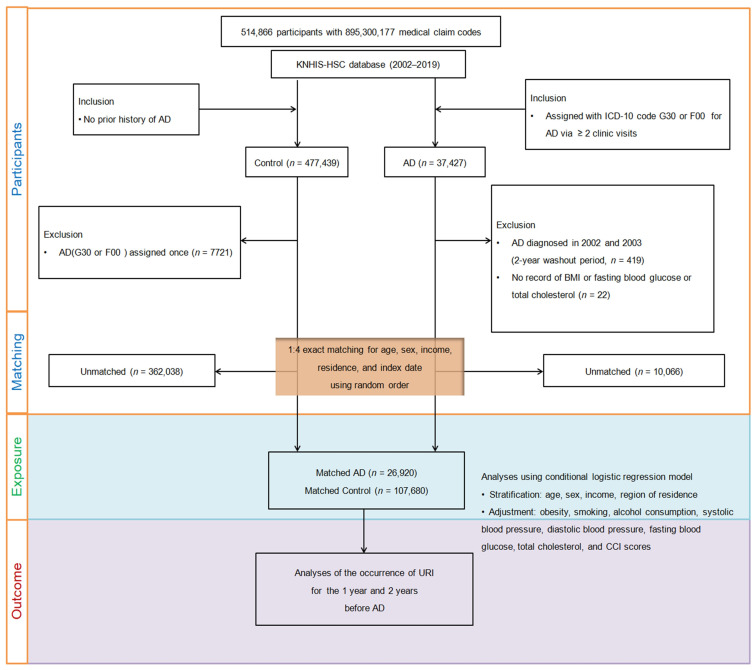
A diagram illustrating the step-by-step process used to select participants for the study. Starting with the initial pool of 514,866 individuals in the Korean National Health Insurance Service—Health Screening Cohort (KNHIS-HSC) database, a meticulous selection process resulted in 26,920 patients diagnosed with Alzheimer’s disease (AD) being matched with 107,680 control participants. Matching was based on age, sex, income, and region of residence.

A 1:4 matching strategy created a control group akin to AD cases in terms of age, sex, income, and region. Random selection from the top of the list ensured an unbiased control group. This process led to the selection of 26,920 AD cases and 107,680 matched controls. The study assessed patients for URI history within 1-year and 2-year periods before the index date in both groups (Figure 1).

### 2.2. Definitions of Upper Respiratory Infection (Exposure) and Alzheimer’s Disease (Outcome)

Patients who sought treatment for URIs (independent variable) during multiple medical visits were categorized based on specific ICD-10 codes, including J00 for acute nasopharyngitis, J02 for acute pharyngitis, and an extension to J069 for acute upper respiratory infection [18]. The frequency of clinic or hospital visits related to URIs was recorded annually. These visits were then accumulated over a two-year period to offer a comprehensive overview of URI frequency and treatment patterns within the participant group. 

Individuals were categorized as having AD (dependent variable) if they received a diagnosis of Alzheimer’s (G30) or dementia in Alzheimer’s (F00). However, this classification was only applied if they had been seen for the same diagnosis on two or more occasions, ensuring the accuracy of the diagnosis [19].

### 2.3. Covariates

Participants were divided into 10 age groups spanning 5-year intervals and grouped into five income categories, from the lowest (class 1) to the highest (class 5). Residential areas were initially sorted into 16 groups based on administrative districts, but were later consolidated into urban areas, including the seven largest Korean cities, while the rest were designated as rural. Similar categorization methods were applied to three variables [20,21]: tobacco smoking (nonsmoker, past smoker, or current smoker); alcohol consumption (<1 time a week, ≥1 time a week); and body mass index (BMI, kg/m^2^), which was categorized as underweight (<18.5), normal weight (≥18.5 to <23), overweight (≥23 to <25), obese I (≥25 to <30), or obese II (≥30) [22]. The study also included health data like systolic and diastolic blood pressures (mmHg), fasting blood glucose levels (mg/dL), and total cholesterol levels (mg/dL) [21]. Additionally, the Charlson Comorbidity Index (CCI) was used to assess the overall disease burden, assigning scores ranging from 0 (no comorbidities) to 29 (multiple comorbidities) based on the severity and number of diseases [23].

### 2.4. Statistical Analyses

We compared the baseline characteristics of the AD and control groups using standardized differences, aiming for an absolute standardized difference of ≤0.20 to achieve balance [24]. For covariates exceeding this threshold, we performed additional adjustments using multivariable logistic regression [24]. To assess the odds ratios (ORs) of URI for AD, we used conditional logistic regression in matched groups based on age, sex, income, and region of residence. Our analyses included crude, model 1 (incorporating smoking status, alcohol drinking, obesity, and CCI scores), and model 2 (which further included fasting blood glucose, total cholesterol, and systolic or diastolic blood pressure) groups. URI treatment instances were categorized into four groups based on treatment frequency, and the corresponding 95% CIs were calculated. Subgroup analyses were conducted considering all covariate variables.

For statistical significance, we employed two-tailed tests and considered results as significant when *p* < 0.05. The statistical analyses were conducted using SAS version 9.4 (SAS Institute Inc., Cary, NC, USA).

## 3. Results

Table 1 provides a summary of the demographic and clinical characteristics for this study, which included 26,920 individuals with AD and 107,680 control participants selected from the database spanning 2002 to 2019. The patient and control groups were meticulously matched, resulting in identical demographic characteristics (age group, sex, economic level, and region of residence) between the AD and control groups, with a standardized difference of 0.00. All other basic characteristics exhibited standardized differences of ≤0.2, indicating no significant disparities between the two groups, except for the CCI scores. Notably, the proportion of patients with a CCI score of 1 or higher was higher in the AD group compared to the controls (68.27% vs. 48.90%).

**Figure 2:**
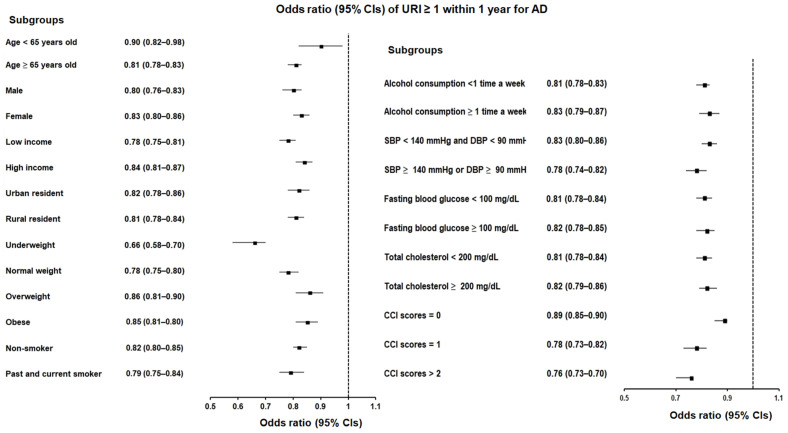
Forest plots illustrating the adjusted odds ratio and corresponding 95% confidence intervals (CIs) for demographic, lifestyle, and comorbid factors in relation to upper respiratory infections (URIs) for incident Alzheimer’s disease (AD) when participants had been diagnosed with URI ≥ 1 within 1 year before the index date.

To bolster the reliability of our findings, we conducted a thorough analysis evaluating URI histories within both the 1-year and 2-year periods before the index date. Consistently, our results indicated a significant inverse relationship between a history of URI and incident AD in both the 1-year and 2-year analyses leading up to the index date. Specifically, when examining ≥1 instances of URI within 1 year, we observed an OR of 0.81 (95% CI = 0.79–0.84, *p* < 0.001), suggesting a 19% reduced likelihood of AD. Subgroup analyses further revealed that this association held true across various demographic categories, remaining significant regardless of age, sex, income, region of residence, CCI scores, hypertension, hyperlipidemia, blood glucose levels, and lifestyle factors such as smoking, alcohol consumption, and obesity (Table 2; Figure 2).

Similarly, participants with either URI ≥ 2 histories or URI ≥ 3 histories within 1 year before the index date exhibited lower odds of AD compared to the control group (0.85 [95% CI = 0.83–0.88, *p* < 0.001]; 0.88 [95% CI = 0.85–0.91, *p* < 0.001], respectively) (Table 3 and Table 4, respectively). Subgroup analyses for both URI histories ≥2 and ≥3 within 1 year consistently demonstrated significant associations across various subgroups, including participants aged ≥ 65 years, males, females, those with low or high income levels, and both urban and rural residents, irrespective of body weight status, smoking or alcohol consumption history, hypertension, hyperlipidemia, or blood glucose levels. However, in the analysis of URI histories ≥2 and ≥3 within a 1-year period, no significant association was found in the <65 years of age subgroup. Moreover, no significant association was observed among participants with CCI scores of 1 in the analysis of URI histories ≥ 3 within a 1-year period.

**Figure 3:**
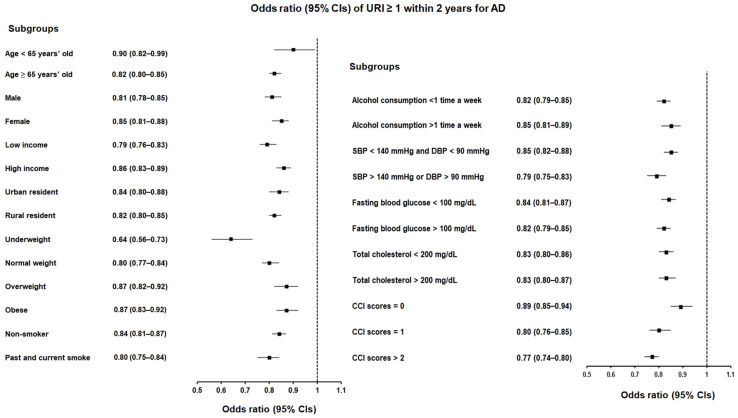
Forest plots illustrating the adjusted odds ratio and corresponding 95% confidence intervals (CIs) for demographic, lifestyle, and comorbid factors in relation to upper respiratory infections (URIs) for incident Alzheimer’s disease (AD) when participants had been diagnosed with URI ≥ 1 within 2 years before the index date.

Similarly, when assessing multiple instances of URI within a 2-year period, the OR was 0.83 (95% CI = 0.81–0.85, *p* < 0.001), indicating a 17% reduced risk of AD compared to the control group (Table 5; Figure 3). The significant association between a history of URI ≥ 1 within 2 years and a lowered likelihood of AD persisted across all subgroup analyses, mirroring the findings from the 1-year period analysis, independent of demographic characteristics, CCI scores, and various other factors (including hypertension; hyperlipidemia; blood glucose levels; and lifestyle factors such as smoking, alcohol consumption, and obesity).

## 4. Discussion

This study thoroughly investigated the potential link between prior URI histories and AD development among Korean adults, analyzing medical records spanning one to two years, with a specific focus on individuals seeking medical treatment at hospitals or clinics for these conditions. Our findings repeatedly demonstrated a notable inverse relationship between the frequency of URI histories and the likelihood of AD. Within a 1-year timeframe, individuals with URI histories (≥1, ≥2, or ≥3 instances) exhibited lower probabilities of AD, with risk reductions of 19% (95% CI = 0.79–0.84), 15% (95% CI = 0.83–0.88), and 12% (95% CI = 0.85–0.91), respectively. Extending our analysis to a 2-year period, we consistently observed a 17% reduction in the likelihood of AD (OR = 0.83), mirroring our 1-year findings. This effect remained robust across diverse demographic groups and even after adjusting for various covariates, including the CCI, hypertension, hyperlipidemia, blood glucose levels, and lifestyle factors. Subgroup analyses further affirmed this association across various demographic and clinical categories, emphasizing its enduring nature. 

In recent decades, the rise of the inflammation–pathogen infection hypothesis has ignited interest in the potential association between infections, specifically URIs, and the initiation and progression of AD [5], with several studies linking pathogens and related inflammatory pathways to AD pathology in human-derived samples and experimental models [25,26,27]. However, there has been no dedicated investigation into the relationship between the URI histories of individuals seeking medical care and their subsequent risk of developing AD. Our results appear to diverge from the findings of two previous population-based cohort studies conducted in the UK and Germany [28,29]. The UK study investigated the association between prior common infections, such as sepsis, pneumonia, lower respiratory tract infections, urinary tract infections, and skin and soft tissue infections, and the onset of dementia [28]. It reported an increased risk of dementia, with a 1.53-fold higher risk for individuals with any infections, a 2.08-fold higher risk of sepsis, and a 1.88-fold higher risk of pneumonia [28]. Meanwhile, the German population-based cohort study explored the link between acute URIs, including COVID-19, and dementia onset [29]. It found that 1.84% of COVID-19 patients and 1.78% of URI patients were diagnosed with dementia after one year, with a non-significant incidence rate ratio of 1.05 (95% CI = 0.85–1.29) [29]. Notably, these two cohort studies did not specify dementia types, but they included various common infections, although they did not focus specifically on AD or URIs [28,29]. They did not achieve a balanced distribution of baseline sociodemographic or health characteristics between the study and control groups, potentially introducing heterogeneity due to demographic disparities and variations in the research groups’ quality [28,29]. In our study, we employed nationwide population-based controls matched using propensity scores to achieve a more accurate balance of baseline characteristics, reducing the study’s heterogeneity and selection bias [30]. Additionally, we conducted multivariate conditional logistic regression analysis to adjust for potential confounding factors. Through these processes, our study indicated that the OR of AD achieved a significant decrease in patients with URI histories. 

A potential explanation for the reduced chance of AD among patients with URIs could be the protective impact of antibiotics and antiviral medications used to treat these infections [31], as our data relied on clinic or hospital visits for URI treatment. Notably, clinical trials are now underway to investigate the effectiveness of antibiotics and antiviral drugs in treating individuals with dementia [13]. In one trial involving individuals with mild to moderate dementia, a combination of antibiotics led to a lesser decline in cognitive function compared to those not taking the drugs [9]. Specifically, a 3-month regimen of doxycycline and rifampin, which can penetrate the blood–brain barrier and are effective against bacteria like Chlamydia pneumoniae, reduced the progressive cognitive decline in AD patients [9]. The mechanism behind this improvement is unlikely to be related to Chlamydia pneumoniae, as there were no differences in its detection between groups using polymerase chain reactions or antibodies [9]. Furthermore, these antibiotics have shown in vitro that they can interfere with the accumulation of the amyloid-β peptide, potentially reducing amyloid deposition in the brains of AD patients [32]. Nonetheless, in another clinical trial where doxycycline or rifampin, either alone or in combination, was administered, no positive effects on cognition or function were observed in individuals with AD [14].

Recent research has demonstrated that whole brain homogenates from individuals with AD exhibit significantly elevated antimicrobial activity compared to age-matched non-AD samples [15]. This heightened antimicrobial activity is closely associated with the levels of amyloid-β within the tissue [15]. Furthermore, the increased antimicrobial action observed in AD brain homogenates can be nullified by immunodepleting them with anti-amyloid-β antibodies [15]. These findings align with the idea that amyloid-β-mediated activity resembles the characteristics of a group of biomolecules collectively referred to as antimicrobial peptides [33,34]. Antimicrobial peptides, also known as host defense peptides, are potent and versatile antibiotics with the ability to target a wide spectrum of pathogens, including Gram-negative and Gram-positive bacteria; mycobacteria; enveloped viruses; fungi; protozoans; and, in some cases [33,34], even transformed or cancerous host cells [35]. Moreover, antimicrobial peptides play a crucial role in immunomodulation, orchestrating cytokine release and contributing to adaptive immune responses [34]. Our results may carefully assume a potential protective role of URI treatment histories in AD development and also suggest that increased frequencies of URI treatments may confer a consistent protective effect against AD.

Several questions remain unanswered. Our study, being observational and retrospective, could not establish a definitive causal link between URI histories or treatments and AD. It also did not delve into the underlying mechanisms. Furthermore, as our study concentrated solely on Korean adults aged 40 and older and primarily utilized Korean health insurance data for exposure assessments, there is a possibility that certain unmeasured confounding factors were overlooked. This could potentially restrict the generalizability of our findings to different populations and demographic categories. Thirdly, it is important to note that the KNHIS-HSC database lacked detailed data pertaining to drug information, the severity of URI and AD, family medical history, personal genetics, and dietary information. As a result, our analysis in this study did not factor in these missing details. These constraints might hinder our capacity to consider potential unmeasured variables that could act as confounding factors, thereby limiting our ability to account for these unmeasured variables in our analysis. Finally, the assessment of OR through age stratification (60–74 and 75+) was not possible, although it could have been intriguing for differentiating early-onset and late-onset AD. This limitation stemmed from the data accessibility constraints within the KNHIS-HSC database. The ownership of the sample cohort data in KNHIS-HSC lies beyond the authors’ control, requiring researchers to access, analyze, and export the outcomes, either by visiting the analysis center or using remote methods.

Nevertheless, this study’s strength lies in its robust cohort, including 26,920 AD patients and 107,680 controls collected meticulously from a nationwide healthcare database. The advantage of this research is its use of a large and representative Korean adult population sample. The comprehensive KNHIS-HSC data provides access to complete medical histories across the country, enhancing the study’s generalizability and precision. The study’s robustness and efforts to minimize bias were enhanced by thorough adjustments for socioeconomic and lifestyle factors, comorbidities, and potential confounding variables. For instance, we addressed the influence of low socioeconomic status as a recognized risk factor for dementia and disparities in dementia diagnosis, where individuals with higher incomes tend to receive earlier diagnoses [36]. Additionally, although AD is more prevalent among females and the elderly [1], and certain factors like current smoking status, lower plasma glucose levels, and BMI have been associated with a higher risk of dementia [37,38], we employed propensity score matching to mitigate potential confounding and selection bias, thereby reducing differences between the study and control groups.

## 5. Conclusions

Our study may hint at an intriguing inverse association between URI histories and the likelihood of AD, particularly among individuals who sought medical treatment at hospitals or clinics for these conditions. This observed relationship persisted over both 1-year and 2-year periods, with those having a history of URI treatments demonstrating reduced probabilities of AD development. Subgroup analyses further reinforced the significance of this association, consistently indicating a lower likelihood of AD development across various demographic and clinical categories. One possible explanation for these results suggests a potential protective role of URI treatment histories in AD development. This is in line with recent research that has explored the potential benefits of therapeutic interventions involving anti-inflammatory, antibiotics, and antiviral medications in the context of AD. Understanding the inverse connection between the treatment histories of URIs and AD may be of importance, not only for enhancing our comprehension of AD’s pathogenesis but also for the development of novel therapeutic and preventive strategies. Further research is needed to explore the underlying mechanisms and to address the limitations of the current study.

## Figures and Tables

**Table 1:** Participant demographics.

Characteristics	AD	Control	Standardized Difference
Age (y), *n* (%)			0.00
40–44	2 (0.01)	8 (0.01)	
45–49	49 (0.18)	196 (0.18)	
50–54	197 (0.73)	788 (0.73)	
55–59	652 (2.42)	2608 (2.42)	
60–64	1510 (5.61)	6040 (5.61)	
65–69	3506 (13.02)	14,024 (13.02)	
70–74	6763 (25.12)	27,052 (25.12)	
75–79	9646 (35.83)	38,584 (35.83)	
80–84	4281 (15.90)	17,124 (15.90)	
85+	314 (1.17)	1256 (1.17)	
Sex, *n* (%)			0.00
Male	11,867 (44.08)	47,468 (44.08)	
Female	15,053 (55.92)	60,212 (55.92)	
Income, *n* (%)			0.00
1 (lowest)	5329 (19.80)	21,316 (19.80)	
2	2857 (10.61)	11,428 (10.61)	
3	3576 (13.28)	14,304 (13.28)	
4	5117 (19.01)	20,468 (19.01)	
5 (highest)	10,041 (37.30)	40,164 (37.30)	
Region of residence, *n* (%)			0.00
Urban	9683 (35.97)	38,732 (35.97)	
Rural	17,237 (64.03)	68,948 (64.03)	
Obesity, *n* (%)			0.12
Underweight	1303 (4.84)	3783 (3.51)	
Normal	10,462 (38.86)	37,524 (34.85)	
Overweight	6511 (24.19)	28,265 (26.25)	
Obese I	7789 (28.93)	34,290 (31.84)	
Obese II	855 (3.18)	3818 (3.55)	
Smoking status, *n* (%)			0.07
Nonsmoker	20,404 (75.79)	81,177 (75.39)	
Past smoker	3440 (12.78)	15,812 (14.68)	
Current smoker	3076 (11.43)	10,691 (9.93)	
Alcohol consumption, *n* (%)			0.07
<1 time a week	18,701 (69.47)	71,438 (66.34)	
≥1 time a week	8219 (30.53)	36,242 (33.66)	
Systolic blood pressure (*n*, %)			0.01
<120 mmHg	6270 (23.29)	23,991 (22.28)	
120–139 mmHg	13,066 (48.54)	54,271 (50.40)	
≥140 mmHg	7584 (28.17)	29,418 (27.32)	
Diastolic blood pressure (*n*, %)			0.02
<80 mmHg	12,828 (47.65)	52,701 (48.94)	
80–89 mmHg	9362 (34.78)	38,004 (35.29)	
≥90 mmHg	4730 (17.57)	16,975 (15.76)	
Fasting blood glucose (*n*, %)			0.12
<100 mg/dL	14,101 (52.38)	59,845 (55.58)	
100–125 mg/dL	8671 (32.21)	35,199 (32.69)	
≥126 mg/dL	4148 (15.41)	12,636 (11.73)	
Total cholesterol (*n*, %)			0.01
<200 mg/dL	15,407 (57.23)	62,080 (57.65)	
200–239 mg/dL	7881 (29.28)	32,301 (30.00)	
≥240 mg/dL	3632 (13.49)	13,299 (12.35)	
CCI score (*n*, %)			0.32
0	8540 (31.72)	55,031 (51.11)	
1	6260 (23.25)	20,888 (19.40)	
≥2	12,120 (45.02)	31,761 (29.50)	
The number of clinic visits for URIs (Mean, SD)			
within 1 year	1.75 (3.98)	1.81 (3.52)	0.02
within 2 years	3.61 (7.05)	3.62 (6.10)	0.07

Abbreviations: AD, Alzheimer’s disease; CCI, Charlson Comorbidity Index; SD, standard deviation; URI, upper respiratory infections.

**Table 2:** Crude and adjusted odds ratios of upper respiratory infections (URIs) for Alzheimer’s disease (AD) when participants are diagnosed with URI ≥ 1 within 1 year before the index date.

	*n* of AD	*n* of Control	Odd Ratios for AD (95% Confidence Interval)					
	(Exposure/Total, %)	(Exposure/Total, %)	Crude ^†^	*p*	Model 1 ^†‡^	*p*	Model 2 ^†§^	*p*
Total (*n* = 134,600)								
No URI	14,771/26,920 (54.9%)	53,352/107,680 (49.6%)	1		1		1	
URI ≥ 1	12,149/26,920 (45.1%)	54,328/107,680 (50.5%)	0.81 (0.79–0.83)	<0.001 *	0.81 (0.79–0.83)	<0.001 *	0.81 (0.79–0.84)	<0.001 *
Age < 65 years old (*n* = 12,050)								
No URI	1365/2410 (56.6%)	5147/9640 (53.4%)	1		1		1	
URI ≥ 1	1045/2410 (43.4%)	4493/9640 (46.6%)	0.88 (0.80–0.96)	0.004 *	0.88 (0.80–0.96)	0.005 *	0.90 (0.82–0.98)	0.022 *
Age ≥ 65 years old (*n* = 122,550)								
No URI	13,406/24,510 (54.7%)	48,205/98,040 (49.2%)	1		1		1	
URI ≥ 1	11,104/24,510 (45.3%)	49,835/98,040 (50.8%)	0.80 (0.78–0.82)	<0.001 *	0.81 (0.78–0.83)	<0.001 *	0.81 (0.78–0.83)	<0.001 *
Men (*n* = 59,335)								
No URI	6915/11,867 (58.3%)	24,900/47,468 (52.5%)	1		1		1	
URI ≥ 1	4952/11,867 (41.7%)	22,568/47,468 (47.5%)	0.79 (0.76–0.82)	<0.001 *	0.80 (0.77–0.83)	<0.001 *	0.80 (0.76–0.83)	<0.001 *
Women (*n* = 75,265)								
No URI	7856/15,053 (52.2%)	28,452/60,212 (47.3%)	1		1		1	
URI ≥ 1	7197/15,053 (47.8%)	31,760/60,212 (52.8%)	0.82 (0.79–0.85)	<0.001 *	0.83 (0.80–0.86)	<0.001 *	0.83 (0.80–0.86)	<0.001 *
Low income (*n* = 58,810)								
No URI	6646/11,762 (56.5%)	23,565/47,048 (50.1%)	1		1		1	
URI ≥ 1	5116/11,762 (43.5%)	23,483/47,048 (49.9%)	0.77 (0.74–0.80)	<0.001 *	0.78 (0.75–0.81)	<0.001 *	0.78 (0.75–0.81)	<0.001 *
High income (*n* = 75,790)								
No URI	8125/15,158 (53.6%)	29,787/60,632 (49.1%)	1		1		1	
URI ≥ 1	7033/15,158 (46.4%)	30,845/60,632 (50.9%)	0.84 (0.81–0.87)	<0.001 *	0.84 (0.81–0.87)	<0.001 *	0.84 (0.81–0.87)	<0.001 *
Urban residents (*n* = 48,415)								
No URI	5529/9683 (57.1%)	20,087/38,732 (51.9%)	1		1		1	
URI ≥ 1	4154/9683 (42.9%)	18,645/38,732 (48.1%)	0.81 (0.77–0.85)	<0.001 *	0.82 (0.78–0.85)	<0.001 *	0.82 (0.78–0.86)	<0.001 *
Rural residents (*n* = 86,185)								
No URI	9242/17,237 (53.6%)	33,265/68,948 (48.3%)	1		1		1	
URI ≥ 1	7995/17,237 (46.4%)	35,683/68,948 (51.8%)	0.81 (0.78–0.83)	<0.001 *	0.81 (0.79–0.84)	<0.001 *	0.81 (0.78–0.84)	<0.001 *
Underweight (*n* = 5,086)								
No URI	807/1303 (61.9%)	1938/3783 (51.2%)	1		1		1	
URI ≥ 1	496/1303 (38.1%)	1845/3783 (48.8%)	0.65 (0.57–0.73)	<0.001 *	0.66 (0.58–0.75)	<0.001 *	0.66 (0.58–0.75)	<0.001 *
Normal weight (*n* = 47,986)								
No URI	5850/10,462 (55.9%)	18,603/37,524 (49.6%)	1		1		1	
URI ≥ 1	4612/10,462 (44.1%)	18,921/37,524 (50.4%)	0.78 (0.74–0.81)	<0.001 *	0.78 (0.75–0.82)	<0.001 *	0.78 (0.75–0.82)	<0.001 *
Overweight (*n* = 34,776)								
No URI	3475/6511 (53.4%)	13,965/28,265 (49.4%)	1		1		1	
URI ≥ 1	3036/6511 (46.6%)	14,300/28,265 (50.6%)	0.85 (0.81–0.90)	<0.001 *	0.86 (0.81–0.91)	<0.001 *	0.86 (0.81–0.91)	<0.001 *
Obese (*n* = 46,752)								
No URI	4639/8644 (53.7%)	18,846/38,108 (49.5%)	1		1		1	
URI ≥ 1	4005/8644 (46.3%)	19,262/38,108 (50.6%)	0.84 (0.81–0.89)	<0.001 *	0.85 (0.81–0.89)	<0.001 *	0.85 (0.81–0.89)	<0.001 *
Non-smoker (*n* = 101,581)								
No URI	10,932/20,404 (53.6%)	39,295/81,177 (48.4%)	1		1		1	
URI ≥ 1	9472/20,404 (46.4%)	41,882/81,177 (51.6%)	0.81 (0.79–0.84)	<0.001 *	0.82 (0.80–0.85)	<0.001 *	0.82 (0.80–0.85)	<0.001 *
Past smoker and current smoker (*n* = 33,019)								
No URI	3839/6516 (58.9%)	14,057/26,503 (53.0%)	1		1		1	
URI ≥ 1	2677/6516 (41.1%)	12,446/26,503 (47.0%)	0.79 (0.75–0.83)	<0.001 *	0.79 (0.75–0.84)	<0.001 *	0.79 (0.75–0.84)	<0.001 *
Alcohol consumption < 1 time a week (*n* = 90,139)								
No URI	10,162/18,701 (54.3%)	34,793/71,438 (48.7%)	1		1		1	
URI ≥ 1	8539/18,701 (45.7%)	36,645/71,438 (51.3%)	0.80 (0.77–0.82)	<0.001 *	0.81 (0.78–0.83)	<0.001 *	0.81 (0.78–0.83)	<0.001 *
Alcohol consumption ≥ 1 time a week (*n* = 44,461)								
No URI	4609/8219 (56.1%)	18,559/36,242 (51.2%)	1		1		1	
URI ≥ 1	3610/8219 (43.9%)	17,683/36,242 (48.8%)	0.82 (0.78–0.86)	<0.001 *	0.83 (0.79–0.87)	<0.001 *	0.83 (0.79–0.87)	<0.001 *
SBP < 140 mmHg and DBP < 90 mmHg (*n* = 93,888)								
No URI	9893/18,540 (53.4%)	36,650/75,348 (48.6%)	1		1		1	
URI ≥ 1	8647/18,540 (46.6%)	38,698/75,348 (51.4%)	0.83 (0.80–0.85)	<0.001 *	0.83 (0.81–0.86)	<0.001 *	0.83 (0.80–0.86)	<0.001 *
SBP ≥ 140 mmHg or DBP ≥ 90 mmHg (*n* = 40,712)								
No URI	4878/8380 (58.2%)	16,702/32,332 (51.7%)	1		1		1	
URI ≥ 1	3502/8380 (41.8%)	15,630/32,332 (48.3%)	0.77 (0.73–0.81)	<0.001 *	0.78 (0.74–0.82)	<0.001 *	0.78 (0.74–0.82)	<0.001 *
Fasting blood glucose < 100 mg/dL (*n* = 73,946)								
No URI	7539/14,101 (53.5%)	28,864/59,845 (48.2%)	1		1		1	
URI ≥ 1	6562/14,101 (46.5%)	30,981/59,845 (51.8%)	0.81 (0.78–0.84)	<0.001 *	0.81 (0.78–0.84)	<0.001 *	0.81 (0.78–0.84)	<0.001 *
Fasting blood glucose ≥ 100 mg/dL (*n* = 60,654)								
No URI	7232/12,819 (56.4%)	24,488/47,835 (51.2%)	1		1		1	
URI ≥ 1	5587/12,819 (43.6%)	23,347/47,835 (48.8%)	0.81 (0.78–0.84)	<0.001 *	0.82 (0.78–0.85)	<0.001 *	0.82 (0.78–0.85)	<0.001 *
Total cholesterol < 200 mg/dL (*n* = 77,487)								
No URI	8490/15,407 (55.1%)	30,819/62,080 (49.6%)	1		1		1	
URI ≥ 1	6917/15,407 (44.9%)	31,261/62,080 (50.4%)	0.80 (0.78–0.83)	<0.001 *	0.81 (0.78–0.84)	<0.001 *	0.81 (0.78–0.84)	<0.001 *
Total cholesterol ≥ 200 mg/dL (*n* = 57,113)								
No URI	6281/11,513 (54.6%)	22,533/45,600 (49.4%)	1		1		1	
URI ≥ 1	5232/11,513 (45.4%)	23,067/45,600 (50.6%)	0.81 (0.78–0.85)	<0.001 *	0.82 (0.79–0.86)	<0.001 *	0.82 (0.79–0.86)	<0.001 *
CCI scores = 0 (*n* = 63,571)								
No URI	4554/8540 (53.3%)	27,753/55,031 (50.4%)	1		1		1	
URI ≥ 1	3986/8540 (46.7%)	27,278/55,031 (49.6%)	0.89 (0.85–0.93)	<0.001 *	0.88 (0.84–0.92)	<0.001 *	0.89 (0.85–0.93)	<0.001 *
CCI score = 1 (*n* = 27,148)								
No URI	3380/6260 (54%)	9947/20,888 (47.6%)	1		1		1	
URI ≥ 1	2880/6260 (46%)	10,941/20,888 (52.4%)	0.77 (0.73–0.82)	<0.001 *	0.77 (0.73–0.82)	<0.001 *	0.78 (0.73–0.82)	<0.001 *
CCI score ≥ 2 (*n* = 43,881)								
No URI	6837/12,120 (56.4%)	15,652/31,761 (49.3%)	1		1		1	
URI ≥ 1	5283/12,120 (43.6%)	16,109/31,761 (50.7%)	0.75 (0.72–0.78)	<0.001 *	0.76 (0.72–0.79)	<0.001 *	0.76 (0.73–0.79)	<0.001 *

Abbreviations: AD, Alzheimer’s disease; URI, upper respiratory infection; SBP, systolic blood pressure; DBP, diastolic blood pressure; CCI, Charlson Comorbidity Index. * Conditional or unconditional logistic regression analysis, significance at *p* < 0.05. ^†^ Stratified model for age, sex, income, and region of residence. ^‡^ Model 1 was adjusted for smoking, alcohol consumption, obesity, and CCI scores. ^§^ Model 2 was adjusted for model 1 plus total cholesterol, systolic blood pressure, diastolic blood pressure, and fasting blood glucose.

**Table 3:** Crude and adjusted odds ratios of upper respiratory infections (URIs) for Alzheimer’s disease (AD) when participants had been diagnosed with URI ≥ 2 within 1 year before the index date.

	*n* of AD	*n* of Control	Odd Ratios for AD (95% Confidence Interval)					
	(Exposure/Total, %)	(Exposure/Total, %)	Crude ^†^	*p*	Model 1 ^†‡^	*p*	Model 2 ^†§^	*p*
Total (*n* = 134,600)								
No URI	18,898/26,920 (70.2%)	71,629/107,680 (66.5%)	1		1		1	
URI ≥ 2	8022/26,920 (29.8%)	36,051/107,680 (33.5%)	0.84 (0.82–0.87)	<0.001 *	0.84 (0.82–0.87)	<0.001 *	0.85 (0.83–0.88)	<0.001 *
Age < 65 years old (*n* = 12,050)								
No URI	1768/2410 (73.4%)	6851/9640 (71.1%)	1		1		1	
URI ≥ 2	642/2410 (26.6%)	2789/9640 (28.9%)	0.89 (0.81–0.99)	0.026 *	0.89 (0.81–0.99)	0.031 *	0.92 (0.83–1.02)	0.111
Age ≥ 65 years old (*n* = 122,550)								
No URI	17,130/24,510 (69.9%)	64,778/98,040 (66.1%)	1		1		1	
URI ≥ 2	7380/24,510 (30.1%)	33,262/98,040 (33.9%)	0.84 (0.81–0.86)	<0.001 *	0.85 (0.82–0.87)	<0.001 *	0.85 (0.82–0.87)	<0.001 *
Men (*n* = 59,335)								
No URI	8645/11,867 (72.9%)	32,640/47,468 (68.8%)	1		1		1	
URI ≥ 2	3222/11,867 (27.2%)	14,828/47,468 (31.2%)	0.82 (0.78–0.86)	<0.001 *	0.83 (0.79–0.87)	<0.001 *	0.83 (0.79–0.87)	<0.001 *
Women (*n* = 75,265)								
No URI	10,253/15,053 (68.1%)	38,989/60,212 (64.8%)	1		1		1	
URI ≥ 2	4800/15,053 (31.9%)	21,223/60,212 (35.3%)	0.86 (0.83–0.89)	<0.001 *	0.87 (0.83–0.90)	<0.001 *	0.87 (0.83–0.90)	<0.001 *
Low income (*n* = 58,810)								
No URI	8358/11,762 (71.1%)	31,508/47,048 (67%)	1		1		1	
URI ≥ 2	3404/11,762 (28.9%)	15,540/47,048 (33%)	0.83 (0.79–0.86)	<0.001 *	0.83 (0.80–0.87)	<0.001 *	0.83 (0.79–0.87)	<0.001 *
High income (*n* = 75,790)								
No URI	10,540/15,158 (69.5%)	40,121/60,632 (66.2%)	1		1		1	
URI ≥ 2	4618/15,158 (30.5%)	20,511/60,632 (33.8%)	0.86 (0.82–0.89)	<0.001 *	0.86 (0.83–0.90)	<0.001 *	0.87 (0.83–0.90)	<0.001 *
Urban residents (*n* = 48,415)								
No URI	6898/9683 (71.2%)	26,292/38,732 (67.9%)	1		1		1	
URI ≥ 2	2785/9683 (28.8%)	12,440/38,732 (32.1%)	0.85 (0.81–0.90)	<0.001 *	0.86 (0.82–0.90)	<0.001 *	0.87 (0.82–0.91)	<0.001 *
Rural residents (*n* = 86,185)								
No URI	12,000/17,237 (69.6%)	45,337/68,948 (65.8%)	1		1		1	
URI ≥ 2	5237/17,237 (30.4%)	23,611/68,948 (34.2%)	0.84 (0.81–0.87)	<0.001 *	0.84 (0.81–0.88)	<0.001 *	0.84 (0.81–0.87)	<0.001 *
Underweight (*n* = 5,086)								
No URI	992/1303 (76.1%)	2564/3783 (67.8%)	1		1		1	
URI ≥ 2	311/1303 (23.9%)	1219/3783 (32.2%)	0.66 (0.57–0.76)	<0.001 *	0.67 (0.58–0.78)	<0.001 *	0.68 (0.59–0.78)	<0.001 *
Normal weight (*n* = 47,986)								
No URI	7440/10,462 (71.1%)	25,037/37,524 (66.7%)	1		1		1	
URI ≥ 2	3022/10,462 (28.9%)	12,487/37,524 (33.3%)	0.81 (0.78–0.85)	<0.001 *	0.82 (0.78–0.86)	<0.001 *	0.82 (0.78–0.86)	<0.001 *
Overweight (*n* = 34,776)								
No URI	4467/6511 (68.6%)	18,714/28,265 (66.2%)	1		1		1	
URI ≥ 2	2044/6511 (31.4%)	9551/28,265 (33.8%)	0.90 (0.85–0.95)	0.000 *	0.90 (0.85–0.95)	0.000 *	0.90 (0.85–0.96)	<0.001 *
Obese (*n* = 46,752)								
No URI	5999/8644 (69.4%)	25,314/38,108 (66.4%)	1		1		1	
URI ≥ 2	2645/8644 (30.6%)	12,794/38,108 (33.6%)	0.87 (0.83–0.92)	<0.001 *	0.88 (0.84–0.93)	<0.001 *	0.87 (0.83–0.92)	<0.001 *
Non-smoker (*n* = 101,581)								
No URI	14,120/20,404 (69.2%)	53,294/81,177 (65.7%)	1		1		1	
URI ≥ 2	6284/20,404 (30.8%)	27,883/81,177 (34.4%)	0.85 (0.82–0.88)	<0.001 *	0.86 (0.83–0.89)	<0.001 *	0.86 (0.83–0.89)	<0.001 *
Past smoker and current smoker (*n* = 33,019)								
No URI	4778/6516 (73.3%)	18,335/26,503 (69.2%)	1		1		1	
URI ≥ 2	1738/6516 (26.7%)	8168/26,503 (30.8%)	0.82 (0.77–0.87)	<0.001 *	0.82 (0.77–0.87)	<0.001 *	0.81 (0.77–0.87)	<0.001 *
Alcohol consumption < 1 time a week (*n* = 90,139)								
No URI	13,065/18,701 (69.9%)	46,993/71,438 (65.8%)	1		1		1	
URI ≥ 2	5636/18,701 (30.1%)	24,445/71,438 (34.2%)	0.83 (0.80–0.86)	<0.001 *	0.84 (0.81–0.87)	<0.001 *	0.84 (0.81–0.87)	<0.001 *
Alcohol consumption ≥ 1 time a week (*n* = 44,461)								
No URI	5833/8219 (71.0%)	24,636/36,242 (68.0%)	1		1		1	
URI ≥ 2	2386/8219 (29.0%)	11,606/36,242 (32.0%)	0.87 (0.82–0.92)	<0.001 *	0.87 (0.83–0.92)	<0.001 *	0.88 (0.83–0.93)	<0.001 *
SBP < 140 mmHg and DBP < 90 mmHg (*n* = 93,888)								
No URI	12,793/18,540 (69.0%)	49,622/75,348 (65.9%)	1		1		1	
URI ≥ 2	5747/18,540 (31.0%)	25,726/75,348 (34.1%)	0.87 (0.84–0.90)	<0.001 *	0.87 (0.84–0.90)	<0.001 *	0.87 (0.84–0.90)	<0.001 *
SBP ≥ 140 mmHg or DBP ≥ 90 mmHg (*n* = 40,712)								
No URI	6105/8380 (72.9%)	22,007/32,332 (68.1%)	1		1		1	
URI ≥ 2	2275/8380 (27.2%)	10,325/32,332 (31.9%)	0.79 (0.75–0.84)	<0.001 *	0.81 (0.76–0.85)	<0.001 *	0.81 (0.77–0.85)	<0.001 *
Fasting blood glucose < 100 mg/dL (*n* = 73,946)								
No URI	9675/14,101 (68.6%)	39,175/59,845 (65.5%)	1		1		1	
URI ≥ 2	4426/14,101 (31.4%)	20,670/59,845 (34.5%)	0.87 (0.83–0.90)	<0.001 *	0.87 (0.83–0.90)	<0.001 *	0.87 (0.84–0.90)	<0.001 *
Fasting blood glucose ≥ 100 mg/dL (*n* = 60,654)								
No URI	9223/12,819 (72%)	32,454/47,835 (67.9%)	1		1		1	
URI ≥ 2	3596/12,819 (28.1%)	15,381/47,835 (32.2%)	0.82 (0.79–0.86)	<0.001 *	0.83 (0.79–0.87)	<0.001 *	0.83 (0.79–0.87)	<0.001 *
Total cholesterol < 200 mg/dL (*n* = 77,487)								
No URI	10,867/15,407 (70.5%)	41,450/62,080 (66.8%)	1		1		1	
URI ≥ 2	4540/15,407 (29.5%)	20,630/62,080 (33.2%)	0.84 (0.81–0.87)	<0.001 *	0.85 (0.81–0.88)	<0.001 *	0.85 (0.81–0.88)	<0.001 *
Total cholesterol ≥ 200 mg/dL (*n* = 57,113)								
No URI	8031/11,513 (69.8%)	30,179/45,600 (66.2%)	1		1		1	
URI ≥ 2	3482/11,513 (30.2%)	15,421/45,600 (33.8%)	0.85 (0.81–0.89)	<0.001 *	0.86 (0.82–0.89)	<0.001 *	0.86 (0.82–0.90)	<0.001 *
CCI score = 0 (*n* = 63,571)								
No URI	5862/8540 (68.6%)	37,144/55,031 (67.5%)	1		1		1	
URI ≥ 2	2678/8540 (31.4%)	17,887/55,031 (32.5%)	0.95 (0.90–1.00)	<0.001 *	0.94 (0.89–0.98)	0.010 *	0.94 (0.89–0.99)	0.013 *
CCI score = 1 (*n* = 27,148)								
No URI	4382/6260 (70%)	13,527/20,888 (64.8%)	1		1		1	
URI ≥ 2	1878/6260 (30%)	7361/20,888 (35.2%)	0.79 (0.74–0.84)	<0.001 *	0.79 (0.74–0.84)	<0.001 *	0.79 (0.74–0.84)	<0.001 *
CCI score ≥ 2 (*n* = 43,881)								
No URI	8654/12,120 (71.4%)	20,958/31,761 (66%)	1		1		1	
URI ≥ 2	3466/12,120 (28.6%)	10,803/31,761 (34%)	0.78 (0.74–0.81)	<0.001 *	0.79 (0.75–0.82)	<0.001 *	0.79 (0.76–0.83)	<0.001 *

Abbreviations: AD, Alzheimer’s disease; URI, upper respiratory infection; SBP, systolic blood pressure; DBP, diastolic blood pressure; CCI, Charlson Comorbidity Index. * Conditional or unconditional logistic regression analysis, significance at *p* < 0.05. ^†^ Stratified model for age, sex, income, and region of residence. ^‡^ Model 1 was adjusted for smoking, alcohol consumption, obesity, and CCI scores. ^§^ Model 2 was adjusted for model 1 plus total cholesterol, systolic blood pressure, diastolic blood pressure, and fasting blood glucose.

**Table 4:** Crude and adjusted odds ratios of upper respiratory infections (URIs) for Alzheimer’s disease (AD) when participants had been diagnosed with URI ≥ 3 within 1 year before the index date.

	*n* of AD	*n* of Control	Odd Ratios for AD (95% Confidence Interval)					
	(Exposure/Total, %)	(Exposure/Total, %)	Crude ^†^	*p*	Model 1 ^†‡^	*p*	Model 2 ^†§^	*p*
Total (*n* = 134,600)								
No URI	21,334/26,920 (79.3%)	82,928/107,680 (77.0%)	1		1		1	
URI ≥ 3	5586/26,920 (20.8%)	24,752/107,680 (23.0%)	0.88 (0.85–0.91)	<0.001 *	0.88 (0.85–0.91)	<0.001 *	0.88 (0.85–0.91)	<0.001 *
Age < 65 years old (*n* = 12,050)								
No URI	1972/2410 (81.8%)	7812/9640 (81.0%)	1		1		1	
URI ≥ 3	438/2410 (18.2%)	1828/9640 (19.0%)	0.95 (0.85–1.07)	0.376	0.95 (0.85–1.07)	0.417	0.98 (0.87–1.11)	0.801
Age ≥ 65 years old (*n* = 122,550)								
No URI	19,362/24,510 (79.0%)	75,116/98,040 (76.6%)	1		1		1	
URI ≥ 3	5148/24,510 (21.0%)	22,924/98,040 (23.4%)	0.87 (0.84–0.90)	<0.001 *	0.88 (0.85–0.91)	<0.001 *	0.88 (0.85–0.91)	<0.001 *
Men (*n* = 59,335)								
No URI	9637/11,867 (81.2%)	37,365/47,468 (78.7%)	1		1		1	
URI ≥ 3	2230/11,867 (18.8%)	10,103/47,468 (21.3%)	0.86 (0.81–0.90)	<0.001 *	0.86 (0.82–0.91)	<0.001 *	0.86 (0.82–0.91)	<0.001 *
Women (*n* = 75,265)								
No URI	11,697/15,053 (77.7%)	45,563/60,212 (75.7%)	1		1		1	
URI ≥ 3	3356/15,053 (22.3%)	14,649/60,212 (24.3%)	0.89 (0.86–0.93)	<0.001 *	0.90 (0.86–0.94)	<0.001 *	0.90 (0.86–0.94)	<0.001 *
Low income (*n* = 58,810)								
No URI	9378/11,762 (79.7%)	36,340/47,048 (77.2%)	1		1		1	
URI ≥ 3	2384/11,762 (20.3%)	10,708/47,048 (22.8%)	0.86 (0.82–0.91)	<0.001 *	0.87 (0.83–0.91)	<0.001 *	0.86 (0.82–0.91)	<0.001 *
High income (*n* = 75,790)								
No URI	11,956/15,158 (78.9%)	46,588/60,632 (76.8%)	1		1		1	
URI ≥ 3	3202/15,158 (21.1%)	14,044/60,632 (23.2%)	0.89 (0.85–0.93)	<0.001 *	0.89 (0.86–0.93)	<0.001 *	0.90 (0.86–0.94)	<0.001 *
Urban residents (*n* = 48,415)								
No URI	7737/9683 (79.9%)	30,125/38,732 (77.8%)	1		1		1	
URI ≥ 3	1,946/9683 (20.1%)	8607/38,732 (22.2%)	0.88 (0.83–0.93)	<0.001 *	0.89 (0.84–0.94)	<0.001 *	0.89 (0.85–0.95)	<0.001 *
Rural residents (*n* = 86,185)								
No URI	13,597/17,237 (78.9%)	52,803/68,948 (76.6%)	1		1		1	
URI ≥ 3	3640/17,237 (21.1%)	16,145/68,948 (23.4%)	0.88 (0.84–0.91)	<0.001 *	0.88 (0.85–0.92)	<0.001 *	0.88 (0.84–0.91)	<0.001 *
Underweight (*n* = 5086)								
No URI	1081/1303 (83.0%)	2941/3783 (77.7%)	1		1		1	
URI ≥ 3	222/1303 (17.0%)	842/3783 (22.3%)	0.72 (0.61–0.84)	<0.001 *	0.73 (0.62–0.86)	<0.001 *	0.74 (0.63–0.87)	0.000 *
Normal weight (*n* = 47,986)								
No URI	8371/10,462 (80.0%)	28,940/37,524 (77.1%)	1		1		1	
URI ≥ 3	2091/10,462 (20.0%)	8584/37,524 (22.9%)	0.84 (0.80–0.89)	<0.001 *	0.85 (0.81–0.90)	<0.001 *	0.85 (0.81–0.9)	<0.001 *
Overweight (*n* = 34,776)								
No URI	5064/6511 (77.8%)	21,698/28,265 (76.8%)	1		1		1	
URI ≥ 3	1447/6511 (22.2%)	6567/28,265 (23.2%)	0.94 (0.89–1.01)	0.081	0.95 (0.89–1.01)	0.109	0.95 (0.89–1.01)	0.095
Obese (*n* = 46,752)								
No URI	6818/8644 (78.9%)	29,349/38,108 (77.0%)	1		1		1	
URI ≥ 3	1826/8644 (21.1%)	8759/38,108 (23.0%)	0.90 (0.85–0.95)	0.000 *	0.90 (0.85–0.96)	<0.001 *	0.90 (0.85–0.95)	0.000 *
Non-smoker (*n* = 101,581)								
No URI	16,021/20,404 (78.5%)	62,034/81,177 (76.4%)	1		1		1	
URI ≥ 3	4383/20,404 (21.5%)	19,143/81,177 (23.6%)	0.89 (0.85–0.92)	<0.001 *	0.89 (0.86–0.93)	<0.001 *	0.90 (0.86–0.93)	<0.001 *
Past smoker and current smoker (*n* = 33,019)								
No URI	5313/6516 (81.5%)	20,894/26,503 (78.8%)	1		1		1	
URI ≥ 3	1203/6516 (18.5%)	5609/26,503 (21.2%)	0.84 (0.79–0.90)	<0.001 *	0.85 (0.79–0.91)	<0.001 *	0.84 (0.78–0.90)	<0.001 *
Alcohol consumption < 1 time a week (*n* = 90,139)								
No URI	14,755/18,701 (78.9%)	54,605/71,438 (76.4%)	1		1		1	
URI ≥ 3	3946/18,701 (21.1%)	16,833/71,438 (23.6%)	0.87 (0.83–0.90)	<0.001 *	0.88 (0.84–0.91)	<0.001 *	0.87 (0.84–0.91)	<0.001 *
Alcohol consumption ≥ 1 time a week (*n* = 44,461)								
No URI	6579/8219 (80.1%)	28,323/36,242 (78.2%)	1		1		1	
URI ≥ 3	1640/8219 (20.0%)	7919/36,242 (21.9%)	0.89 (0.84–0.95)	<0.001 *	0.90 (0.84–0.95)	<0.001 *	0.90 (0.85–0.96)	<0.001 *
SBP < 140 mmHg and DBP < 90 mmHg (*n* = 93,888)								
No URI	14,528/18,540 (78.4%)	57,651/75,348 (76.5%)	1		1		1	
URI ≥ 3	4012/18,540 (21.6%)	17,697/75,348 (23.5%)	0.90 (0.87–0.94)	<0.001 *	0.91 (0.87–0.94)	<0.001 *	0.90 (0.87–0.94)	<0.001 *
SBP ≥ 140 mmHg or DBP ≥ 90 mmHg (*n* = 40,712)								
No URI	6806/8380 (81.2%)	25,277/32,332 (78.2%)	1		1		1	
URI ≥ 3	1574/8380 (18.8%)	7055/32,332 (21.8%)	0.83 (0.78–0.88)	<0.001 *	0.84 (0.79–0.89)	<0.001 *	0.84 (0.79–0.90)	<0.001 *
Fasting blood glucose < 100 mg/dL (*n* = 73,946)								
No URI	10,988/14,101 (77.9%)	45,569/59,845 (76.2%)	1		1		1	
URI ≥ 3	3113/14,101 (22.1%)	14,276/59,845 (23.9%)	0.90 (0.87–0.95)	<0.001 *	0.91 (0.87–0.95)	<0.001 *	0.90 (0.87–0.95)	<0.001 *
Fasting blood glucose ≥ 100 mg/dL (*n* = 60,654)								
No URI	10,346/12,819 (80.7%)	37,359/47,835 (78.1%)	1		1		1	
URI ≥ 3	2473/12,819 (19.3%)	10,476/47,835 (21.9%)	0.85 (0.81–0.90)	<0.001 *	0.86 (0.82–0.90)	<0.001 *	0.86 (0.82–0.90)	<0.001 *
Total cholesterol < 200 mg/dL (*n* = 77,487)								
No URI	12,262/15,407 (79.6%)	48,018/62,080 (77.4%)	1		1		1	
URI ≥ 3	3145/15,407 (20.4%)	14,062/62,080 (22.7%)	0.88 (0.84–0.91)	<0.001 *	0.88 (0.85–0.92)	<0.001 *	0.88 (0.84–0.92)	<0.001 *
Total cholesterol ≥ 200 mg/dL (*n* = 57,113)								
No URI	9072/11,513 (78.8%)	34,910/45,600 (76.6%)	1		1		1	
URI ≥ 3	2441/11,513 (21.2%)	10,690/45,600 (23.4%)	0.88 (0.84–0.92)	<0.001 *	0.89 (0.84–0.93)	<0.001 *	0.89 (0.84–0.93)	<0.001 *
CCI score = 0 (*n* = 63,571)								
No URI	6648/8540 (77.9%)	42,930/55,031 (78%)	1		1		1	
URI ≥ 3	1892/8540 (22.2%)	12,101/55,031 (22%)	1.01 (0.96–1.07)	0.731	1.00 (0.94–1.05)	0.932	1.00 (0.95–1.06)	0.969
CCI score = 1 (*n* = 27,148)								
No URI	4968/6260 (79.4%)	15,755/20,888 (75.4%)	1		1		1	
URI ≥ 3	1292/6260 (20.6%)	5133/20,888 (24.6%)	0.80 (0.75–0.86)	<0.001 *	0.80 (0.74–0.85)	<0.001 *	0.80 (0.75–0.86)	<0.001 *
CCI score ≥ 2 (*n* = 43,881)								
No URI	9718/12,120 (80.2%)	24,243/31,761 (76.3%)	1		1		1	
URI ≥ 3	2402/12,120 (19.8%)	7518/31,761 (23.7%)	0.80 (0.76–0.84)	<0.001 *	0.81 (0.77–0.85)	<0.001 *	0.81 (0.77–0.86)	<0.001 *

Abbreviations: AD, Alzheimer’s disease; URI, upper respiratory infection; SBP, systolic blood pressure; DBP, diastolic blood pressure; CCI, Charlson Comorbidity Index. * Conditional or unconditional logistic regression analysis, significance at *p* < 0.05. ^†^ Stratified model for age, sex, income, and region of residence. ^‡^ Model 1 was adjusted for smoking, alcohol consumption, obesity, and CCI scores. ^§^ Model 2 was adjusted for model 1 plus total cholesterol, systolic blood pressure, diastolic blood pressure, and fasting blood glucose.

**Table 5:** Crude and adjusted odds ratios of upper respiratory infections (URIs) for Alzheimer’s disease (AD) when participants had been diagnosed with URI ≥ 1 within 2 years before the index date.

	*n* of AD	*n* of Control	Odd Ratios for AD (95% Confidence Interval)					
	(Exposure/Total, %)	(Exposure/Total, %)	Crude ^†^	*p*	Model 1 ^†‡^	*p*	Model 2 ^†§^	*p*
Total (*n* = 134,600)								
No URI	10,056/26,920 (37.4%)	35,412/107,680 (32.9%)	1		1		1	
URI ≥ 1	16,864/26,920 (62.6%)	72,268/107,680 (67.1%)	0.82 (0.80–0.84)	<0.001 *	0.82 (0.80–0.85)	<0.001 *	0.83 (0.81–0.85)	<0.001 *
Age < 65 years old (*n* = 12,050)								
No URI	967/2410 (40.1%)	3587/9640 (37.2%)	1		1		1	
URI ≥ 1	1443/2410 (59.9%)	6053/9640 (62.8%)	0.88 (0.81–0.97)	0.008 *	0.88 (0.81–0.97)	0.009 *	0.90 (0.82–0.99)	0.030 *
Age ≥ 65 years old (*n* = 122,550)								
No URI	9089/24,510 (37.1%)	31,825/98,040 (32.5%)	1		1		1	
URI ≥ 1	15,421/24,510 (62.9%)	66,215/98,040 (67.5%)	0.82 (0.79–0.84)	<0.001 *	0.82 (0.80–0.85)	<0.001 *	0.82 (0.80–0.85)	<0.001 *
Men (*n* = 59,335)								
No URI	4898/11,867 (41.3%)	17,129/47,468 (36.1%)	1		1		1	
URI ≥ 1	6969/11,867 (58.7%)	30,339/47,468 (63.9%)	0.80 (0.77–0.84)	<0.001 *	0.81 (0.78–0.85)	<0.001 *	0.81 (0.78–0.85)	<0.001 *
Women (*n* = 75,265)								
No URI	5158/15,053 (34.3%)	18,283/60,212 (30.4%)	1		1		1	
URI ≥ 1	9895/15,053 (65.7%)	41,929/60,212 (69.6%)	0.84 (0.81–0.87)	<0.001 *	0.84 (0.81–0.88)	<0.001 *	0.85 (0.81–0.88)	<0.001 *
Low income (*n* = 58,810)								
No URI	4595/11,762 (39.1%)	15,778/47,048 (33.5%)	1		1		1	
URI ≥ 1	7167/11,762 (60.9%)	31,270/47,048 (66.5%)	0.79 (0.75–0.82)	<0.001 *	0.80 (0.76–0.83)	<0.001 *	0.79 (0.76–0.83)	<0.001 *
High income (*n* = 75,790)								
No URI	5461/15,158 (36.0%)	19,634/60,632 (32.4%)	1		1		1	
URI ≥ 1	9697/15,158 (64.0%)	40,998/60,632 (67.6%)	0.85 (0.82–0.88)	<0.001 *	0.86 (0.83–0.89)	<0.001 *	0.86 (0.83–0.89)	<0.001 *
Urban residents (*n* = 48,415)								
No URI	3858/9683 (39.8%)	13,689/38,732 (35.3%)	1		1		1	
URI ≥ 1	5825/9683 (60.2%)	25,043/38,732 (64.7%)	0.83 (0.79–0.86)	<0.001 *	0.83 (0.80–0.87)	<0.001 *	0.84 (0.80–0.88)	<0.001 *
Rural residents (*n* = 86,185)								
No URI	6198/17,237 (36.0%)	21,723/68,948 (31.5%)	1		1		1	
URI ≥ 1	11,039/17,237 (64.0%)	47,225/68,948 (68.5%)	0.82 (0.79–0.85)	<0.001 *	0.83 (0.80–0.86)	<0.001 *	0.82 (0.80–0.85)	<0.001 *
Underweight (*n* = 5086)								
No URI	595/1303 (45.7%)	1305/3783 (34.5%)	1		1		1	
URI ≥ 1	708/1303 (54.3%)	2478/3783 (65.5%)	0.63 (0.55–0.71)	<0.001 *	0.64 (0.56–0.73)	<0.001 *	0.64 (0.56–0.73)	<0.001 *
Normal weight (*n* = 47,986)								
No URI	4016/10,462 (38.4%)	12,444/37,524 (33.2%)	1		1		1	
URI ≥ 1	6446/10,462 (61.6%)	25,080/37,524 (66.8%)	0.80 (0.76–0.83)	<0.001 *	0.81 (0.77–0.84)	<0.001 *	0.80 (0.77–0.84)	<0.001 *
Overweight (*n* = 34,776)								
No URI	2347/6511 (36.1%)	9233/28,265 (32.7%)	1		1		1	
URI ≥ 1	4164/6511 (64.0%)	19,032/28,265 (67.3%)	0.86 (0.81–0.91)	<0.001 *	0.87 (0.82–0.92)	<0.001 *	0.87 (0.82–0.92)	<0.001 *
Obese (*n* = 46,752)								
No URI	3098/8644 (35.8%)	12,430/38,108 (32.6%)	1		1		1	
URI ≥ 1	5546/8644 (64.2%)	25,678/38,108 (67.4%)	0.87 (0.83–0.91)	<0.001 *	0.88 (0.83–0.92)	<0.001 *	0.87 (0.83–0.92)	<0.001 *
Non-smoker (*n* = 101,581)								
No URI	7304/20,404 (35.8%)	25,691/81,177 (31.7%)	1		1		1	
URI ≥ 1	13,100/20,404 (64.2%)	55,486/81,177 (68.4%)	0.83 (0.80–0.86)	<0.001 *	0.84 (0.81–0.87)	<0.001 *	0.84 (0.81–0.87)	<0.001 *
Past smoker and current smoker (*n* = 33,019)								
No URI	2752/6516 (42.2%)	9721/26,503 (36.7%)	1		1		1	
URI ≥ 1	3764/6516 (57.8%)	16,782/26,503 (63.3%)	0.79 (0.75–0.84)	<0.001 *	0.80 (0.75–0.84)	<0.001 *	0.80 (0.75–0.84)	<0.001 *
Alcohol consumption < 1 time a week (*n* = 90,139)								
No URI	6842/18,701 (36.6%)	22,768/71,438 (31.9%)	1		1		1	
URI ≥ 1	11,859/18,701 (63.4%)	48,670/71,438 (68.1%)	0.81 (0.78–0.84)	<0.001 *	0.82 (0.79–0.85)	<0.001 *	0.82 (0.79–0.85)	<0.001 *
Alcohol consumption ≥ 1 time a week (*n* = 44,461)								
No URI	3214/8219 (39.1%)	12,644/36,242 (34.9%)	1		1		1	
URI ≥ 1	5005/8219 (60.9%)	23,598/36,242 (65.1%)	0.83 (0.79–0.88)	<0.001 *	0.84 (0.80–0.88)	<0.001 *	0.85 (0.81–0.89)	<0.001 *
SBP < 140 mmHg and DBP < 90 mmHg (*n* = 93,888)								
No URI	6632/18,540 (35.8%)	24,110/75,348 (32.0%)	1		1		1	
URI ≥ 1	11,908/18,540 (64.2%)	51,238/75,348 (68.0%)	0.84 (0.82-0.87)	<0.001 *	0.85 (0.82-0.88)	<0.001 *	0.85 (0.82–0.88)	<0.001 *
SBP ≥ 140 mmHg or DBP ≥ 90 mmHg (*n* = 40,712)								
No URI	3424/8380 (40.9%)	11,302/32,332 (35.0%)	1		1		1	
URI ≥ 1	4956/8380 (59.1%)	21,030/32,332 (65.0%)	0.78 (0.74–0.82)	<0.001 *	0.79 (0.75–0.83)	<0.001 *	0.79 (0.75–0.83)	<0.001 *
Fasting blood glucose < 100 mg/dL (*n* = 73,946)								
No URI	11,302/32,332 (35.0%)	18,960/59,845 (31.7%)	1		1		1	
URI ≥ 1	21,030/32,332 (65.0%)	40,885/59,845 (68.3%)	0.84 (0.81–0.87)	<0.001 *	0.84 (0.81–0.87)	<0.001 *	0.84 (0.81–0.87)	<0.001 *
Fasting blood glucose ≥ 100 mg/dL (*n* = 60,654)								
No URI	5033/12,819 (39.3%)	16,452/47,835 (34.4%)	1		1		1	
URI ≥ 1	7786/12,819 (60.7%)	31,383/47,835 (65.6%)	0.81 (0.78–0.84)	<0.001 *	0.82 (0.79–0.85)	<0.001 *	0.82 (0.79–0.85)	<0.001 *
Total cholesterol < 200 mg/dL (*n* = 77,487)								
No URI	5765/15,407 (37.4%)	20,479/62,080 (33.0%)	1		1		1	
URI ≥ 1	9642/15,407 (62.6%)	41,601/62,080 (67.0%)	0.82 (0.79–0.85)	<0.001 *	0.83 (0.80–0.86)	<0.001 *	0.83 (0.80–0.86)	<0.001 *
Total cholesterol ≥ 200 mg/dL (*n* = 57,113)								
No URI	4291/11,513 (37.3%)	14,933/45,600 (32.8%)	1		1		1	
URI ≥ 1	7222/11,513 (62.7%)	30,667/45,600 (67.3%)	0.82 (0.79–0.86)	<0.001 *	0.83 (0.79–0.86)	<0.001 *	0.83 (0.80–0.87)	<0.001 *
CCI score = 0 (*n* = 63,571)								
No URI	3096/8540 (36.3%)	18,625/55,031 (33.8%)	1		1		1	
URI ≥ 1	5444/8540 (63.8%)	36,406/55,031 (66.2%)	0.90 (0.86–0.94)	<0.001 *	0.89 (0.85–0.93)	<0.001 *	0.89 (0.85–0.94)	<0.001 *
CCI score = 1 (*n* = 27,148)								
No URI	2248/6260 (35.9%)	6457/20,888 (30.9%)	1		1		1	
URI ≥ 1	4012/6260 (64.1%)	14,431/20,888 (69.1%)	0.80 (0.75–0.85)	<0.001 *	0.80 (0.75–0.85)	<0.001 *	0.80 (0.76–0.85)	<0.001 *
CCI score ≥ 2 (*n* = 43,881)								
No URI	4712/12,120 (38.9%)	10,330/31,761 (32.5%)	1		1		1	
URI ≥ 1	7408/12,120 (61.1%)	21,431/31,761 (67.5%)	0.76 (0.73–0.79)	<0.001 *	0.77 (0.73–0.80)	<0.001 *	0.77 (0.74–0.80)	<0.001 *

Abbreviations: AD, Alzheimer’s disease; URI, upper respiratory infection; SBP, systolic blood pressure; DBP, diastolic blood pressure; CCI, Charlson Comorbidity Index. * Conditional or unconditional logistic regression analysis, significance at *p* < 0.05. ^†^ Stratified model for age, sex, income, and region of residence. ^‡^ Model 1 was adjusted for smoking, alcohol consumption, obesity, and CCI scores. ^§^ Model 2 was adjusted for model 1 plus total cholesterol, systolic blood pressure, diastolic blood pressure, and fasting blood glucose.

## Data Availability

All data are available from the database of National Health Insurance Sharing Service (NHISS) https://nhiss.nhis.or.kr/ (accessed on 1 March 2023). NHISS allows access to all of this data for any researcher who promises to follow the research ethics at some processing charge. If you want to access the data of this article, you can download it from the website after promising to follow the research ethics.
